# Virus removal by high-efficiency air (HEPA) filters and filtration capacity enhancement by nanotextiles: a pilot study

**DOI:** 10.1007/s12223-024-01137-4

**Published:** 2024-02-14

**Authors:** Daniela Obitková, Milan Mráz, Emil Pavlík

**Affiliations:** 1 Department of Health Care Disciplines and Population Protection, Faculty of Biomedical Engineering, Czech Technical University in Prague , Náměstí Sítná 3105 Kladno, Czech Republic; 2 Faculty of Biomedical Engineering, Czech Technical University in Prague, Náměstí Sítná 3105 Kladno, Czech Republic; 3 Medical Immunology and Microbiology Institute, 1st Medical Faculty, Charles University, Studničkova 7, Prague, Czech Republic

**Keywords:** Coronavirus, HEPA filter, Indoor air, Nanotextile, Respiratory virus

## Abstract

Portable household air purifiers are widely used devices designed to maintain a high-quality level of indoor air. Portable air purifiers equipped with the high-efficiency air (HEPA) filter served 100 h in a household space occupied by two adults without any symptoms of respiratory tract infection. The main objective of the study was to determine microbial contamination on the HEPA filter and to investigate if the selected nanotextile monolayer made of polyamide 6 (PA6) nanofibers can capture potential microorganisms when installed downstream of the HEPA filter as the final filtration medium. Samples were taken from the inlet and outlet surfaces. Samples from the nanotextile were collected in the same manner as from the HEPA filter. QIAStat DX® 1.0 Analyzer using the Respiratory SARS CoV-2 Panel multiplex PCR detection system was selected for microorganism detection. Adenovirus was detected on the inlet surface of the HEPA filter. The outlet surface of the filter contained no viruses included in the Respiratory SARS CoV-2 Panel portfolio. The nanotextile monolayer was replaced twice during the 100 h of operation, so three pieces were used and all contained coronavirus 229 E. Coronavirus 229 E was then detected in the nasopharynx of one of the members of the household as well. It may be assumed that the selected nanotextile is capable of capturing a virus of a small size.

## Introduction

Infectious diseases, their transmission, and their spread have posed a threat to human beings from ancient times to the present. The wide range of routes of infectious disease transmission, especially those caused by airborne pathogens, has to be considered. We study microbial contamination of air filters installed in different air conditioning systems and portable air purifiers and evaluate their role in the spread of airborne pathogens. Each air-conditioning system or portable air purifier contains an efficient air filter that should capture particles dispersed in the circulating air.

Today many air conditioning systems and portable air purifiers use HEPA filters. Air filters (including HEPA filters) are usually made of patented filter media. Fibers are made of fiberglass, expanded polytetrafluoroethylene (Perry et al. 2016), polyester, or polypropylene. Advanced materials such as polylactic acid (PLA) fibers are currently being investigated (Wang et al. 2015). Other biodegradable materials could also be of interest and are also in focus (Lippi et al. 2022). Filtration media are usually pleated within a framework containing some support elements or may be support-free. HEPA filters are relatively well-defined in Europe and in the USA. As defined by the Institute of Environmental Sciences and Technology (IEST, USA) according to the standards for air filter efficiency evaluation and testing (IEST-RP-CC001.3 and MIL-STD-282), HEPA filters must capture a minimum of 99.97% of particles at 0.3 µm in size. In Europe, the mandatory standard is EN 1822:2019 which defines HEPA filter as having a minimum capture rate of 99.995% for particles of 0.3 µm in size. ("EN 1822–1:2019" 2019). Particles of this size are the most difficult to capture and are therefore considered to be the most penetrating particle size (MPPS) (Lee and Liu 1980).

Microbes differ from solid particles in several ways, such as density, the presence of hydrophobic capsules or slime layers, and in having flagella that enable motility. The rod-shaped nature of different bacteria may also be a factor in the difference in filtration efficiency from predictions based on particle size. However, it has been shown that particle filtration models can be reliably converted to microbial particles (Kowalski et al. 1999). Bacteria generally range in size from 0.1 to 10 µm, while viruses, although they can form clusters or be bound to other particles, are typically 25–400 nm in diameter (Hogan et al. 2005).

It was suggested that viruses could penetrate the filtration media of the HEPA filter. An example could be the severe acute respiratory syndrome virus SARS CoV-1 spread on board of the commercial aircraft. Olsen et al. described the pattern of spread of SARS-CoV-1 on board (Olsen et al. 2003). Other research groups reported that the HEPA filter did not capture the viruses of smaller sizes optimally (Helmbuch et al. 2007).

Recently, the nanotechnology field has had a great impact on various fields such as healthcare or the environment, especially in the capture of gaseous and particle pollutants (Ravichandran et al. 2012) (Orlando et al. 2021). Among the nanotechnology products, nanofibers are one of the unique materials. Nanofibers made by electrospinning technique have unique properties in air filtration (Sundarrajan et al. 2014). In healthcare, nanotextiles made of nanofibers can enhance the filtration effect and protection effect for example in face masks (El-Atab et al. 2021). During the COVID-19 pandemic, nanofiber textiles proved to have significant potential to capture viruses, especially face masks (Naragund and Panda 2022). Electro-spun nanofiber-based nanotextiles could also be used for water filtration (Lev et al. 2010). The progress in nano fiber-based nanotextiles promises significant air filtration improvement of any use (Alia and Ain 2020).

In this study, we have decided to investigate potential microbial contamination in a real-world situation. The promising features of nanotextile inspired our research group to investigate virus collection by a nanotextile monolayer in a home air purifier. A nanotextile of electrospun polyamide 6 nanofibers overlaid with a non-woven polyester fabric was placed downstream of the standard HEPA filter of a portable air purifier. The main objective of the study was to evaluate the microbial contamination of this HEPA filter and the monolayer of nanotextile behind the filter. For the detection of microbial contamination, we chose the QIAStat DX® multiplex RT-PCR analyzer, which is designed for clinical use as we wanted to take advantage of its ability to detect a broad spectrum of infectious agents that cause respiratory tract infections. We focused primarily on the detection of respiratory viruses. Increasing the filtration capacity of HEPA filters could be of particular interest in times of dangerous viral disease pandemics.

## Material and methods

### Chemicals

Chemicals were obtained from standard suppliers (P-Lab, Czech Republic, Penta, Czech Republic) and were of the highest purity. To avoid unwanted DNA contamination, Termi-DNA-tor spray (Dynex, Czech Republic) was used. A sterile saline solution (0.9% NaCl Braun, Germany) was used as the sampling solution

### Material specification

A portable home air purifier produced in the Czech Republic equipped with a standard HEPA filter was used as a model device. The selected air purifier was purchased from a retail chain and is suitable for household use only. The maximum air flow rate of 145 m^3^/h was used in the experiment and the air ionization option was turned off. The device was placed in a room with a floor area of 63 m^2^ and a volume of 138.6 m^3^ air. The household was occupied by two subjectively healthy adults with no clinical signs of respiratory infection. The experiment was performed during the spring season of 2021 at room temperatures ranging from 20 to 25 °C. The nanotextile sample was obtained from the Nanotex Ltd., Czech Republic. The substance of the nanomaterial is polyamide 6 (PA6) with a porosity of 50 nm and a fibre thickness of 100–500 nm. The heat resistance reaches 126 °C. The nanotextile itself is attached to a polyester non-woven fabric. The dry swabs were taken from the inlet and outlet surface of the HEPA filter and from the nanotextile surface facing the outlet surface of the HEPA filter. For this purpose, the polyester swabs were used (Inset CZ Ltd. Czech Republic). Other plastic laboratory equipment as sterile tubes necessary for sampling were supplied by P-Lab, Czech Republic.

### Sampling

The HEPA filter and the monolayers of nanotextile were aseptically removed from the air conditioner. The filter media were then packed in plastic bags and immediately transported to the laboratory. In the laboratory, all samples were collected in a biohazard box (BSL 2). Dry swabs were taken from the inlet and outlet surface of the HEPA filter and the inlet surface of the nanotextile monolayer. Polyester swabs were used (Inset CZ Ltd.) for this purpose. The swabs were transferred into 1 mL of sterile saline solution (0.9% NaCl Braun). According to the manufacturer’s recommendations, 200 µL of this solution was applied to the QIAStat DX® Respiratory SARS CoV-2 Panel cartridge.

### Multiplex PCR tests

The QIAStat DX® Analyzer 1.0 with the Respiratory SARS CoV-2 Panel (Genetica Ltd., Czech Republic) is a fully automated device used for multiplex RT PCR analyses. The selected respiratory panel contains a wide range of target viral pathogens. The full list of target samples of the respiratory panel includes influenza A, influenza A (subtype H1N1/2009), influenza A (subtype H1), influenza A (subtype H3), influenza B, coronavirus 229E, coronavirus HKU1, coronavirus NL63, coronavirus OC43, SARS-CoV-2, parainfluenza virus 2, parainfluenza virus 3, parainfluenza virus 4, respiratory syncytial virus A/B, human metapneumovirus A/B, adenovirus, bocavirus, and rhinovirus/enterovirus. Also included are three bacterial targets *— Mycoplasma pneumoniae*, *Legionella pneumophilla, Bordetella pertusis*.

## Results

### Experiment arrangement

**Fig. 1:**
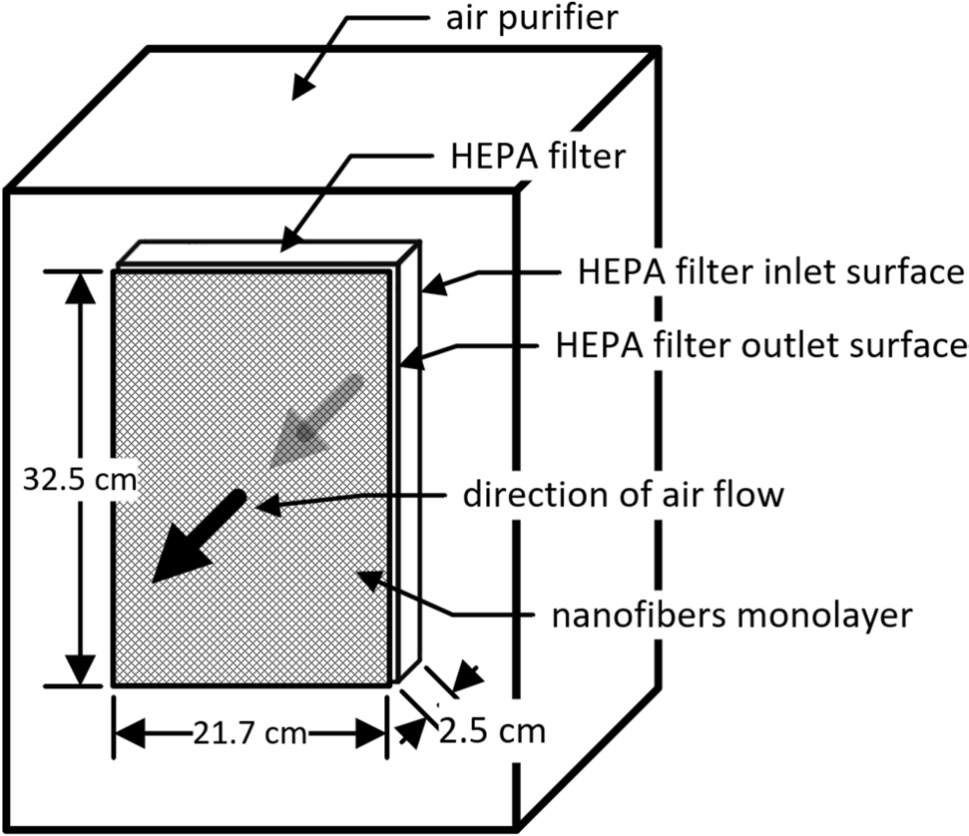
The experimental setup

The household air purifier used as a model device in this work consisted of an inlet section where a fan driven by an electric motor draws air into the device. The air then leaves the device through a HEPA filter. The air filter is covered by a paper frame and placed in the plastic cell of the air conditioner. The plastic cell provided sufficient space to place a single monolayer behind the exit face of the HEPA filter as the last filter media. The size of the nanotextile was proportional to the size of the HEPA filter to reduce leakage of filtered air. Experimental setup — air enters the inlet surface of the HEPA filter and continues into the filter layers. The air then exits the HEPA filter and passes through a monolayer of nanofibers. The air then exits the unit. The dimensions of the HEPA filter and nanofiber layer are 32.5 × 15.6 × 2.5 cm. The dimensions of the whole air purifier were 39.6 cm (length), 21.7 cm (width), and 50.2 cm (height). The experimental setup is depicted in Fig. 1.

In this configuration, the air purifier was used for 100 h. The monolayer of nanotextile was replaced three times to prevent clogging of the nanotextile pores and degradation of the experiment. At the very end, we obtained one HEPA filter and three individual nanotextile sheets of rectangular shape and the same size as the HEPA filter. Then a sample was taken from the surface of the HEPA filter and the nanotextile monolayer. Each surface — HEPA inlet, HEPA outlet, nanotextiles 1 to 3 — was wiped with dry polyester swabs. The swabs were rinsed in 1 mL of sterile saline solution, yielding 5 samples.

### RT PCR

The QIAStat DX® Analyzer 1.0 with the Respiratory SARS CoV-2 Panel (Genetica Ltd, CZ) is designed primarily for clinical use. In our experiment, we decided to use this instrument because of its simple use and wide range of target viral pathogens. The cartridges produced for the respiratory panel provide two possible ways of sample applications. The whole swab or liquid sample can be inserted into the cartridge. For our experiment, we chose to apply the liquid sample exactly according to the manufacturer’s instructions. Each of our five samples was applied to one cartridge so that the QIAStat DX® Analyzer 1.0 automated cycler performed 5 separate cycles. The sixth sample was then obtained by swabbing the throat of a member of the experimental household who provided it voluntarily (volunteer). This sample had its own cartridge and its own analysis run. The detection results are published in a qualitative manner. The measurement report contains the information — detected/not detected, the results are published in a qualitative way only. Quantification of the viral load is not available.

The RT PCR procedure revealed that the inlet surface of the HEPA filter contained an adenovirus. It did not penetrate the filter, so the outlet surface did not contain any virus included in the portfolio of the respiratory panel. Coronavirus 229E was not detected on either the HEPA filter inlet surface or the HEPA filter outlet surface. It was only detected on the monolayer of the nanotextile. It was detected on all three experimental parts of the nanotextile. Coronavirus 229E was detected in the throat of a volunteer from the experimental household. The RT PCR results are summarized in the following Table 1.

**Table 1:** Results of virus detection

Type of the filter	Detected pathogen
HEPA FILTER inlet	Adenovirus
HEPA filter outlet	NONE
Nanotextile 1	Coronavirus 229E
Nanotextile 2	Coronavirus 229E
Nanotextile 3	Coronavirus 229E
Volunteer	Coronavirus 229E

## Discussion

Viruses are the biggest problem for air filters. Smaller viruses, which are usually smaller than 300 nm, are not completely eliminated by HEPA filters. Consistent with this claim, the nanotextile monolayer did indeed contain coronavirus 229E. It has been shown previously that HEPA filters do not capture viruses adequately. This may be due to several factors. Firstly, viruses can enter the air filter through defects in the material itself, caused for example by pleating of the individual layers of the filter medium. Pinhole leaks may be another cause of filtration capacity failure (Harstad and Filler 2007). Other studies on HEPA filter efficiency also report limited capture of viral particles by HEPA filters (Helmbuch et al. 2007). HEPA filters operate at the level of a HEPA filter that filters 99.97% of MMPs. The results of the present study are quite consistent with our previous studies revealing poor virus capture in HEPA filters. In particular, coronavirus 229E penetrated the HEPA filter in our previous study of a home air purifier equipped by a HEPA filter (Obitková and Pavlík 2019). Adenovirus is also significantly small, with a diameter in the range of 70 to 100 nm. This suggests that it can penetrate through the filter. The occurrence of this virus on the inlet surface of the filter may be due to the droplet mode of transmission of this virus (Baron 1996). Droplets are removed from filtered air by HEPA filter more efficiently than for example aerosols. The COVID-19 pandemic prompted more rapid development of personal protection measures, including the development of face masks and respirators made of nanomaterials. Nanotextiles are a promising means of air filtration. Our selected monolayer of nanotextiles captured coronavirus 229E. This pathogenic virus is small, suggesting that nanotextiles could be a sufficient means of air filtration. The nanotextile used in this experiment has several key properties. The 50 nm pore size covers the diameter of most human viral pathogens. If the nanotextile served only as a sieve, no viruses of our interest would penetrate the chosen monolayer of nanotextile. On the other hand, the small pores of the nanotextile may also be an obvious disadvantage. Nanotextiles could not be used as the sole filter medium due to the clogging of the pores by dust or similar larger particles present in the filtered air. Air filters made of nanomaterials designed as a nanofibrous monolayer with a microfibrous support can have a significantly higher efficiency in eliminating submicron aerosols (Podgórski et al. 2006). From this perspective, our chosen nanotextile can significantly increase the filtration efficiency of a standard HEPA filter.

As mentioned above, larger particles are usually a challenge for nanomaterial air filters due to surface loading. The filtered particles from the circulated air are trapped only on the surface of the nanofiber filter. They do not penetrate deep into the filter medium as in the case of conventional air filters. In our last experiment, we encountered surface loading of the nanotextile monolayer after 100 h of filtration (Obitková and Pavlík 2019). Moreover, some viruses have short lifespan in the external environment — adenoviruses can survive on the fabric for less than 24 h, and influenza A and B viruses can survive on selected surfaces for 24–48 h (Pirtle and Beran 1991). Therefore, we decided to replace the nanotextile monolayer three times with a replacement interval of approximately 33 h. From a technical point of view, we are unable to estimate the lifetime of the nanomaterial because no information was provided by the manufacturer of the nanomaterial used in the experiment.

The Respiratory SARS CoV-2 Panel real-time PCR detection kit running on the QIAStat DX® 1.0 platform was a suitable choice to cover most of the respiratory viruses we were primarily looking for. The Respiratory Virus Panel is designed to cover all major causative agents of upper and lower respiratory tract infections. The operation of the analyzer is simple and the results obtained were sufficient for our purpose. The RT PCR technique provides very reliable detection of viral nucleic acid. For future research, the quantification of viral load on the nanotextile monolayer or the viability of the detected viruses could be investigated. Although the minimum infectious dose for virus-induced diseases is very low, we believe that quantification of HEPA filter-transmitted viruses could at least be of interest. Furthermore, the QIAStat SARS CoV-2 panel used did not allow differentiation between different types of adenoviruses. To find a specific type, we would use an adenovirus-specific analysis kit in any type of RT PCR cycler.

The HEPA filter was contaminated with adenovirus. In the spring season, when respiratory infections are more common, a second member of the experimental household may have been the source of the adenovirus. The chosen multiplex RT PCR analyzer does not allow the specification of adenovirus, so we cannot accurately determine the type of adenovirus. We can only assume that the detected adenovirus originated from the respiratory tract, as adenoviral conjunctivitis is more typical for the summer season and adenoviral infections of the digestive or urinary tract are spread through urine or stool (Goering et al. 2018). On the other hand, the source of coronavirus 229 E is well known and was found in the nasopharynx of one of the members of the experimental household. This household member may have been in the incubation period of the upper respiratory tract infection.

The current experimental setup was designed to be easy to install with a small number of samples. In comparison to other studies of portable air purifiers equipped with HEPA filters provided indoors — home or school, (Rodríguez et al. 2021; Myers et al. 2022), our experiment is unique in that swabs of the HEPA filter surface and the nanotextile monolayer demonstrate the presence of viral contamination. In their study, Rodriguez et.al. conducted a study on the effectiveness of portable air purifiers in eliminating SARS-CoV-2 in several households in different cities and demonstrated an 80% elimination efficiency of air purifiers. Lindsay et al. also simulated the effectiveness of an air purifier in a single room (Lindsley et al. 2021). We know that the experiment from one household could not be able to show objective information. Contamination of the HEPA filter or the monolayer of nanotextiles could have occurred during the replacement of the nanotextile or during laboratory procedures on both the HEPA filter and the nanomaterial. Despite the simple experimental setup and small sample size, we have provided valuable data in investigating the ability of nanomaterials to trap viruses and increase the efficiency of conventional air filters.

## Conclusion

Human pathogens affecting the upper and lower respiratory tract were searched for on a HEPA filter and a nanotextile monolayer in a home portable air purifier. The results showed the presence of adenovirus on the inlet surface of the HEPA filter and coronavirus 229 E on the nanotextile monolayer. This may indicate that viruses may be trapped by the nanotextile. A proven source of coronavirus 229 E was present in the experimental household and the chosen nanotextile was able to capture the coronavirus.
